# Validating intramyocardial bone marrow stem cell therapy in combination with coronary artery bypass grafting, the PERFECT Phase III randomized multicenter trial: study protocol for a randomized controlled trial

**DOI:** 10.1186/1745-6215-13-99

**Published:** 2012-07-02

**Authors:** Peter Donndorf, Alexander Kaminski, Gudrun Tiedemann, Guenther Kundt, Gustav Steinhoff

**Affiliations:** 1 Department of Cardiac Surgery, Reference and Translation Centre for Cardiac Stem cell therapy, University of Rostock, Schillingallee 35, Rostock, 18057, Germany; 2 Institute for Biostatistics and Informatics in Medicine and Ageing Research, University of Rostock, Rostock, Germany

**Keywords:** CABG, Intramyocardial, Ischemic heart disease, Trial, Stem cell

## Abstract

**Background:**

For the last decade continuous efforts have been made to translate regenerative cell therapy protocols in the cardiovascular field from ‘bench to bedside’. Successful clinical introduction, supporting safety, and feasibility of this new therapeutic approach, led to the initiation of the German, Phase III, multicenter trial - termed the PERFECT trial (ClinicalTrials.gov Identifier: NCT00950274), in order to evaluate the efficacy of surgical cardiac cell therapy on left ventricular function.

**Methods/Design:**

The PERFECT trial has been designed as a prospective, randomized, double-blind, placebo controlled, multicenter trial, analyzing the effect of intramyocardial CD 133^+^ bone marrow stem cell injection in combination with coronary artery bypass grafting on postoperative left ventricular function. The trial includes patients aged between 18 and 79 years presenting with a coronary disease with indication for surgical revascularization and reduced global left ventricular ejection fraction as assessed by cardiac magnet resonance imaging. The included patients are treated in the chronic phase of ischemic cardiomyopathy after previous myocardial infarction.

**Discussion:**

Patients undergoing coronary artery bypass grafting in combination with intramyocardial CD133^+^ cell injection will have a higher LV ejection fraction than patient who undergo CABG alone, measured 6 months after the operation.

**Trial registration:**

ClinicalTrials.gov Identifier: NCT00950274

## Background

Ten years ago, after promising preclinical results [1,2], cardiac stem cell application for regenerative purposes was introduced in the fields of interventional cardiology [3] and cardiac surgery [4] for the treatment of acute myocardial infarction and chronic ischemic heart disease. Since intrinsic myocardial regeneration takes places but is reduced during a normal life span [5], it is conceivable that, in addition to revascularization procedures, elderly patients in particular, forming the vast majority of cardiologic and cardio-surgical patients, might profit from attempts aimed at stimulating these regenerative processes.

In our own group the capability of human CD133^+^ stem cells to restore cardiac function after myocardial cryoinjury was tested utilizing immunodeficient mouse models. Compared to CD133^+^ progenitor cells from the cord blood, CD133^+^ cells derived from the bone marrow were found to have beneficial effects on both post-injury angiogenesis and survival as well as myocardial contractility [6]. Moreover, by tracking of human DNA, the persistence of progenitor cell progeny in the heart after intramyocardial injection could be shown. As other groups, we did not see direct myocardial differentiation of these cells, although differentiation towards endothelial phenotypes was clearly shown. Therefore it is questionable whether transdifferentiation of bone marrow stem cells from hematopoietic linages to functional cardiomyocytes (the hypothesis on which regenerative strategies in the cardiovascular field were once based) truly takes place. The reported functional improvement is more likely due to the support of angiogenesis and arteriogenesis in the ischemic myocardium and the protection of cardiomyocytes from apoptotic cell death [7].

All these findings set the path for a clinical pilot trial (Phase I) and subsequent controlled trial (Phase II) studying the impact of intramyocardial injection of CD133^+^ bone marrow stem cells in combination with coronary artery bypass grafting (CABG) surgery in chronic ischemic heart disease [8]. The clear and reproducible beneficial effects of CD133^+^ cell therapy regarding functional recovery of the ischemic myocardium and the absence of any relevant side effects of intramyocardial cell injection in the preclinical setting, provided the basis for the shift from ‘bench to bedside’. After the first reports of successful stem cell delivery to the injured heart, the feasibility and safety of this new therapeutic strategy could be proven for intracoronary [9] as well as surgical intramyocardial [10] cell delivery, thereby focusing on the treatment of ischemic heart disease. Over 1,000 patients with either recent myocardial infarction or chronic ischemic heart failure have been treated so far by means of interventional cardiology and cardiac surgery [11].

However, the clinical efficacy of cardiac stem cell application remains an open question even today. In order to answer this question in terms of surgical stem cell therapy, a randomized, double-blind, placebo controlled, Phase III multicenter clinical trial - PERFECT, Intramyocardial Trans**P**lantation of Bon**E** Ma**R**row Stem Cells **F**or Improv**E**ment of Post-Infarct Myo**C**ardial Regenera**T**ion in Addition to CABG Surgery - was launched. The following null-hypothesis will be tested: ‘Patients undergoing CABG & CD133^+^ cell injection do not have a different LV ejection fraction than patient who undergoing CABG alone, measured 6 months after the operation’. Currently, the PERFECT trial is recruiting patients.

The PERFECT trial has been approved by both independent local ethics committees as well as the Paul Erlich Institute representing a competent federal authority in accordance with German legal requirements. All procedures will be performed in accordance with the principles of Good Clinical Practice (GCP) and the Declaration of Helsinki.

## Methods/Design

### Study design and clinical endpoints

The PERFECT trial (ClinicalTrials.gov Identifier: NCT00950274) has been designed as a prospective, randomized, double-blind, placebo controlled, multicenter trial, applying GCP standards. It is aimed at addressing the issues mentioned above by providing evidence for the effects of cardiac stem cell therapy in combination with CABG surgery on ventricular function as well as patients’ clinical outcomes and quality of life. Patients between 18 and 79 years presenting with a coronary disease with indication for CABG and reduced global left ventricular ejection fraction (LVEF) as assessed by cardiac magnet resonance imaging (MRI) are included. For detailed trial inclusion and exclusion criteria see Table 1. Every patient being referred to CABG and presenting with echocardiographic reduced LVEF after myocardial infarction is screened for possible trial inclusion. Patient screening is performed by physicians as well as study nurses and final decision on CABG indication is taken by a cardiac surgeon. Pre- and postoperative LVEF assessment by cardiac MRI is performed in one core lab facility and analyzed by a radiologist. Final decision on trial inclusion is made based on the preoperative MRI result and after written inform consent has been provided. The day of CABG surgery is defined as the time point zero.

**Table 1:** Major patient inclusion and exclusion criteria for the PERFECT trial (ClinicalTrials.gov Identifier: NCT00950274)

**Patient inclusion criteria**	**Exclusion criteria**
Coronary artery disease with indication for CABG and previous myocardial infarction	Emergency operation
LVEF between 25% and 50% measured by cardiac MRI	Acute myocardial infarction within last 2 weeks
Akinetic/hypokinetic/hibernating left ventricular areas localized by cardiac stress MRI	Resuscitation in combination with ventricular arrhythmias within the last 14 days before treatment
Absence of any moderate to severe valvular heart disease requiring concomitant valve replacement or reconstruction	Active cancer, organ transplantation, end-stage renal disorder
Informed consent of the patient	Infection (CRP ≥ 20 mg/L, temperature ≥38.5 °C)
Age between 18 and 79 years	Contraindication for MRI

CRP, c-reactive protein; LVEF, left ventricular ejection fraction; MRI, magnet resonance imaging.

### Primary endpoint

Left ventricular ejection fraction at rest, measured by MRI (time frame: 6 months)

### Secondary endpoints

Change in LVEF as assessed by MRI and echocardiography (time frame: preoperatively, early postoperatively (discharge), and 6 months)

Regional contractility in the area of interest (AOI)/Change in left ventricular end systolic diameter (LVESD), left ventricular end diastolic diameter (LVEDD) as assessed by echocardiography (time frame: preoperatively, early postoperatively (discharge), 6 months)

Physical exercise capacity determined by 6-min walk test (time frame: preoperatively, early postoperatively (discharge), 6 months)

NYHA and CCS class (time frame: preoperatively, early postoperatively (discharge), 6 months)

MACE (cardiac death, myocardial infarction, secondary intervention/reoperation, ventricular arrhythmia) (time frame: 6 months)

QoL (Quality of life)-score: Minnesota Living with Heart Failure Questionnaire, SF36 Questionnaire, EQ-5D Questionnaire (time frame: preoperatively, 3 months, 6 months)

The following null hypothesis will be tested: ‘Patients undergoing CABG & CD133^+^ cell injection do not have a different LV ejection fraction than patient who undergoing CABG alone, measured 6 months after the operation’. Considerations for determining the sample size of the primary efficacy parameter LVEP at 6 months postoperatively, measured by MRI at rest, were carried out on the basis of the results of a previous efficacy pilot trial. The stratification of the primary analysis by center is neglected in the sample size calculation. Instead of the analysis of covariance (ANCOVA) to be used in the primary analysis, the two-sample *t* test scenario with equal variances is considered.

Sample size determination was done under the assumption of a two-sided type I error (α) at 5% and a type II error (β) at 10% (that is, a power at 90%). The scenario of a difference of about 4 to 5 is considered as a clinical relevant difference. With a difference of 4.5 and a standard deviation of 7.5, at least *n* = 60 patients per group are necessary and with an additional 15% drop-out rate a total of at least 142 patients will be randomized.

Patients will be randomized in a 1:1 ratio to undergo CABG surgery in conjunction with either intramyocardial injection of autologous CD133-enriched bone marrow cells or placebo. The randomization procedure is stratified by study site and as randomization procedure the permuted block design within strata is used. The study treatment is randomly assigned in advance according to the randomization list held and stored by a Biostatistician and using sealed envelopes provided to the bone-marrow isolation laboratory. According to the information randomization is to be carried out and CD133^+^ cell isolation product or placebo will be produced for the current patient.

Concealment is realized in a way that only members of the team of the bone-marrow isolation laboratory will have access to the randomization code. All members have signed an agreement, stating that the randomization code will be kept confidential and no person outside members of the team of bone-marrow isolation laboratory will have access to the code.

### Cell preparation

Isolation of CD133^+^ stem cells from the aspirated bone marrow before CABG surgery is performed for all study sites in one core lab facility under good manufacturing practice (GMP) conditions using the CliniMACS CD133 System (Miltenyi Biotec GmbH, Bergisch Gladbach, Germany). Random allocation will be performed in the cell production facility, so that neither the patient nor the surgeon nor any of the personnel involved in follow-up examinations will know whether the cell product or placebo was administered.

A total of 0.5 to 5 × 10^6^ CD133^+^ cells are prepared for injection. The numerical difference to the doses used in Phase I and II studies are due to different counting methods.

### Surgical stem cell injection during CABG

Intraoperative injections are performed into the target regions after completion of the distal graft anastomoses just before release of the aortic cross-clamp, utilizing standardized 1 mL syringes. CD 133^+^ cells are suspended in 5 mL physiological saline containing 10% autologous serum. In control patients, equivalent placebo injections containing physiological saline and 10% autologous serum are performed. To visualize the target areas for cell injections appointed by preoperative MRI analysis, a segmental map of the left ventricular myocardium is brought to the operating theater and targeted myocardial regions are marked for optimal orientation of the surgeon. Moreover this kind of documentation allows exact analysis of segmental changes in ventricular function during follow-up in the context of cell injection target areas.

### Statistical methods

The trial will be conducted in several study sites. Therefore centers will be considered as possible prognostic factor influencing the outcome.

With regard to possible baseline and study site effects, the two-sided hypothesis for the continuous primary efficacy variable LVEF at 6 months postoperatively will be assessed using analysis of covariance (ANCOVA) adjusting for baseline LVEF and study sites.

Secondary efficacy variables will be analyzed in a strictly explorative way. If *P* values are computed, no adjustment for multiple testing will be made and they will be interpreted in the exploratory sense. Similarly, confidence intervals computed will be interpreted as interval estimates for presence or absence of effects in the study data.

In order to check differences between the treatment groups for particular secondary endpoints (death, myocardial infarction, need of reintervention) an unadjusted survival analysis with Kaplan-Meier estimations will be performed using the logrank test.

Two different sets of patients will be defined to perform analyses. A ‘Full Analysis Set’ (FAS) following the principle of intent-to-treat (ITT) will include every patient as randomized and compare the patients according to the group to which they were randomly allocated, regardless of patients’ compliance, or withdrawal from the study. It is the most cautious approach and so minimizes type 1 error, helps preserve prognostic balance in the study arms and allows for the greatest generalizability.

Secondly, an analysis of all per protocol treated patients will be performed. The ‘Per Protocol Set’ (PPS) sample will consist of all subjects from the FAS group without any major protocol violation.

First and foremost, confirmatory analyses on primary efficacy variable will be performed on the FAS patients but a secondary analysis will also be performed based upon the PPS, to assess the sensitivity of the analysis to the choice of analysis population. The ITT analysis will be considered as the primary one.

The safety population will comprise all patients randomized into the study, in which each patient’s treatment is as taken on the study. Safety evaluations will be performed on the safety population. Adverse events and serious adverse events will be listed summed by occurrence, severity, outcome, and causal relationship to treatment and will be descriptively compared between the two treatment groups using Fishers exact test.

All statistical tests will be conducted using a two-sided test with α = 0.05.

The statistical analysis of study results will be performed according to the CPMP guidelines for ‘Biostatistical methodology in clinical trials in applications for marketing authorizations for medical products’ and the ICH guideline ‘Statistical principles for clinical trials’.

## Discussion

The PERFECT trial, representing the first Phase III trial for evaluation of intramyocardial bone marrow stem cell therapy during CABG surgery, was initiated in 2009. Based on solid preclinical evidence gained from preclinical results demonstrating the capability of bone marrow stem cells to partially restore cardiac function after ischemic damage in animal models and clinical results from Phase I and II trials an evidence based RCT (randomized controlled trial) is mandatory to make a step forward in evaluating cardiac cell therapy as an relevant therapeutic tool. However, if the Phase III trial results support cardiac cell therapy as an effective therapeutic option in chronic ischemic heart disease, further efforts will most likely need to be taken to answer questions regarding the exact mechanisms of stem cell migration, differentiation and myocardial regeneration, in order to optimize cell therapy protocols. Additionally the optimal time point of cell application is still unclear. The current concept for optimal timing of cardiac cell therapy after ischemic myocardial injury is characterized by a ‘window of opportunity’ representing a time point with the inflammatory response, the healing and the scarring balanced in a most favorable way for the requirements of homing, survival, and differentiation of therapeutically applied stem cells. Currently, this time point seems to be within the first month after myocardial infarction for the treatment with bone marrow stem cells [12]. But the clinical evidence regarding the optimal time point is sparse, especially concerning the setting of chronic ischemic heart disease. For the PERFECT trial a minimal time frame of 2 weeks between the underlying myocardial infarction and cell therapy is required. This provides acceptable tissue conditions for bypass surgery and the primary inflammatory response is likely to have passed.

Only by a combined approach consisting of both, research in the lab and consequent clinical translation, can stem cell therapy become a truly standardized, therapeutic ‘tool’ in the field of regenerative medicine. Therefore, and in addition to the clinical endpoints addressed, intensive applied basic research is carried out concomitantly to the PERFECT trial. Isolated CD133^+^ stem cells from the patient’s bone marrow are characterized by fluorescence activated cell sorting (FACS) analysis and subpopulations are described for further classification and standardization of the cell product. Vitality and proliferation assays are performed and angiogenic potency is assessed utilizing CFU-EC (colony forming unit- endothelial cell) analysis in order to verify the regenerative capacity of CD133^+^ cells. To learn more about the possible intrinsic regenerative response mechanisms to operative and/or ischemic stress, peripheral blood is analyzed at different points in time in the perioperative course for markers of angiogenesis (VEGF, FGF), inflammatory response (IL-6, IL-8, TNF-α), endothelial activation (sE-selectin), and heart failure (myoglobin). Additionally, perioperative mobilization of certain stem cell sub-populations, like endothelial progenitor cells, is assessed. In this way the PERFECT trial, besides providing evidence regarding the impact of surgical stem cell therapy on patients’ functional and clinical outcome, helps to further standardize the GMP produced, autologous cell product and to answer questions raised by the basic research.

Furthermore, the authors believe that exact, critical, and standardized pre-procedural evaluation of left ventricular function as well as examination of regional myocardial viability, preferably by cardiac MRI, form another key issue for reaching possible therapeutic efficacy by means of cardiac cell therapy. However, novel imaging modalities like speckle tracking by two-dimensional strain echocardiography might offer useful additional tools for planning and evaluation of cardiac cell therapy in the future [13].

### Trial status

Recruiting.

## Abbreviations

CABG, Coronary Artery Bypass Grafting; CCS, Canadian Cardiovascular Society; CD, Cluster of Differentiation; CFU-EC, Colony forming Unit- Endothelial Cell; GCP, Good Clinical Practice; MACE, Major Adverse Cardiovascular Event; MRI, Magnet Resonance Imaging; NYHA, New York Heart Association; LVEF, Left Ventricular Ejection Fraction; LVEDD, Left Ventricular End Diastolic Diameter; LVESD, Left Ventricular End Systolic Diameter; QoL, Ouality of Life.

## Competing interests

The author declared that they have no competing interests.

## Authors’ contributions

PD drafted the manuscript and carried out parts of the preclinical research. AK made substantial contributions to the concept and design of the manuscript and carried out parts of the preclinical research. GT contributed to conception and design of the PERFECT trial and revised the manuscript critically. GK is in charge of the statistical analysis and described the respective statistical methods in the manuscript. GS contributed to conception and design of the PERFECT trial and acts as the primary investigator of the PERFECT trial. All authors read and approved the final manuscript.

## Authors’ information

GS acts as the primary investigator of the PERFECT trial.

## Sources of funding

The PERFECT trial is supported by the German Federal Ministry of Education and Research (Grant Nr. 0312138A) as well as Miltenyi Biotec GmbH.
